# A New Antisense Phosphoryl Guanidine Oligo-2′-O-Methylribonucleotide Penetrates Into Intracellular Mycobacteria and Suppresses Target Gene Expression

**DOI:** 10.3389/fphar.2019.01049

**Published:** 2019-09-19

**Authors:** Yulia V. Skvortsova, Elena G. Salina, Ekaterina A. Burakova, Oksana S. Bychenko, Dmitry A. Stetsenko, Tatyana L. Azhikina

**Affiliations:** ^1^Shemyakin and Ovchinnikov Institute of Bioorganic Chemistry, Russian Academy of Sciences, Moscow, Russia; ^2^Bach Institute of Biochemistry, Research Center of Biotechnology of the Russian Academy of Sciences, Moscow, Russia; ^3^Institute of Chemical Biology and Fundamental Medicine, Siberian Branch of the Russian Academy of Sciences, Novosibirsk, Russia; ^4^Faculty of Physics, Novosibirsk State University, Novosibirsk, Russia

**Keywords:** multidrug resistance, tuberculosis, antibacterial agents, antisense oligonucleotides, cellular uptake, RNase H, macrophages

## Abstract

The worldwide spread of multidrug-resistant *Mycobacterium tuberculosis* strains prompted the development of new strategies to combat tuberculosis, one of which is antisense therapy based on targeting bacterial mRNA by oligonucleotide derivatives. However, the main limitation of antisense antibacterials is poor cellular uptake because of electrostatic charge. Phosphoryl guanidine oligo-2′-*O*-methylribonucleotides (2′-OMe PGOs) are a novel type of uncharged RNA analogues with high RNA affinity, which penetrate through the bacterial cell wall more efficiently. In this study, we investigated the uptake and biological effects of 2′-OMe PGO in mycobacteria. The results indicated that 2′-OMe PGO specific for the alanine dehydrogenase-encoding *ald* gene inhibited the growth of *Mycobacterium smegmatis* and downregulated *ald* expression at both the transcriptional and translational levels through an RNase H-independent mechanism, showing higher biological activity than its phosphorothioate oligonucleotide counterpart. Confocal microscopy revealed that the anti-*ald* 2′-OMe PGO was taken up by intracellular mycobacteria residing in RAW 264.7 macrophages without exerting toxic effects on eukaryotic cells, indicating that 2′-OMe PGO was able to efficiently cross two cellular membranes. In addition, 2′-OMe PGO inhibited the transcription of the target *ald* gene in *M. smegmatis*-infected macrophages. Thus, we demonstrated, for the first time, a possibility of targeting gene expression and inhibiting growth of intracellular mycobacteria by antisense oligonucleotide derivatives. Strong antisense activity and efficient uptake of the new RNA analogue, 2′-OMe PGO, by intracellular microorganisms revealed here may promote the development of novel therapeutic strategies to treat TB and prevent the emergence of drug-resistant mycobacterial strains.

## Introduction

Tuberculosis (TB), a chronic infectious disease caused by *Mycobacterium tuberculosis*, is responsible for nearly 2 million fatalities annually, and in 2017, 10 million new TB cases were documented (WHO report, 2018). Only 5–10% of infected individuals develop progressive lung disease, whereas the rest have latent infection without symptoms, and to date, more than 25% of the total human population are believed to be asymptomatic carriers of the pathogen (Veatch and Kaushal, 2018), which, however, can be reactivated, indicating high risk of developing active TB in a large proportion of the population. Furthermore, the disease can be refractory to treatment because the widespread use of conventional antibiotics has led to the emergence of multidrug-resistant (MDR) and extremely drug resistant (XDR) *M. tuberculosis* strains, which accounts for a decreased success recovery rate: according to the latest WHO data, it is 52% for MDR-TB and 28% for XDR-TB vs. 83% for TB. Therefore, alternative therapeutic options overcoming limitations of traditional antimicrobial drugs are urgently required.

Antisense therapy is an approach to treat bacterial infections using short (15–25 nucleotides) single-stranded oligonucleotides, often chemically modified to gain stability in biological media (Sully and Geller, 2016). Antisense oligonucleotides (ASOs) act by forming a complementary duplex with their target mRNA through specific sites (Zamecnik and Stephenson, 1978; Uhlmann and Peyman, 1990) and thus can modulate gene expression in a sequence-selective manner by inhibiting translation through either steric block (i.e., physical arrest of ribosomal elongation) or RNase H-mediated degradation of the mRNA strand hybridized with the ASO (Kurreck, 2003). ASOs can be introduced into cells by gymnotic (“naked”) uptake without the aid of lipophilic carriers, which is convenient and eliminates possible toxic effects of transfection agents (Dowdy, 2017; Shen and Corey, 2018).

Antisense antibacterials can be designed to target conserved mRNA regions critical for the life cycle or antibiotic resistance of the pathogen, while not leading to the emergence of drug-resistant strains (Altman, 2014; Penchovsky and Traykovska, 2015). The approach is considered very promising in combating *M. tuberculosis* infection (AlMatar et al., 2017; Hegarty and Stewart, 2018), and several attempts have been made to use ASOs modified with phosphorothioate (PS) or peptide nucleic acids (PNAs) to target essential mycobacterial genes (reviewed in Hegarty and Stewart, 2018). However, the use of ASOs as antibacterial agents is limited, in particular, by their poor uptake into either extracellular or, especially, intracellular bacteria (Xue et al., 2018), and the majority of them have been designed to work in eukaryotic cell systems (Juliano, 2016), with the exception of peptide conjugates with phosphorodiamidate morpholino oligonucleotides (PMOs or morpholinos) (Daly et al., 2017) or, to a lesser extent, PNAs (Good et al., 2001). Relatively poor efficiency of cellular uptake for common ASO derivatives without specific transfection agents or delivery systems could be, at least partly, attributed to their large net negative charge (Fokina et al., 2017), which prevents their penetration through hydrophobic cell membranes.

Phosphoryl guanidine oligo-2′-*O*-methylribonucleotides (2′-OMe PGOs) are a novel type of charge-neutral nucleic acid analogues developed by the Stetsenko group (Kupryushkin et al., 2014). Previous studies indicate that phosphoryl guanidine oligonucleotides are structurally similar to natural oligodeoxynucleotides and demonstrate improved nuclease resistance (Lomzov et al., 2019; Su et al., 2019). Our preliminary experiments have revealed that 2′-OMe PGOs combine the benefits of several ASO types as they are chemically and biologically stable similar to deoxy PGOs, possess high RNA affinity comparable with that of oligo-2′-*O*-methylribonucleotides, and, as morpholinos, lack negative charge (Kupryushkin et al., 2014; Fokina et al., 2018). Therefore, we hypothesized that 2′-OMe PGOs may have improved cellular uptake and functional activity in inhibiting translation of mycobacterial genes.

Here, we report that 2′-OMe PGO reduced the growth of mycobacteria in culture and inhibited translation of the target mRNA more effectively than its PS counterpart. Furthermore, 2′-OMe PGO showed the ability to penetrate into intracellular macrophage-residing mycobacteria without the aid of transfection agents and suppress target gene expression, suggesting its potential as a novel and effective antimycobacterial agent.

## Materials and Methods

### Oligonucleotides

**Figure 1:**
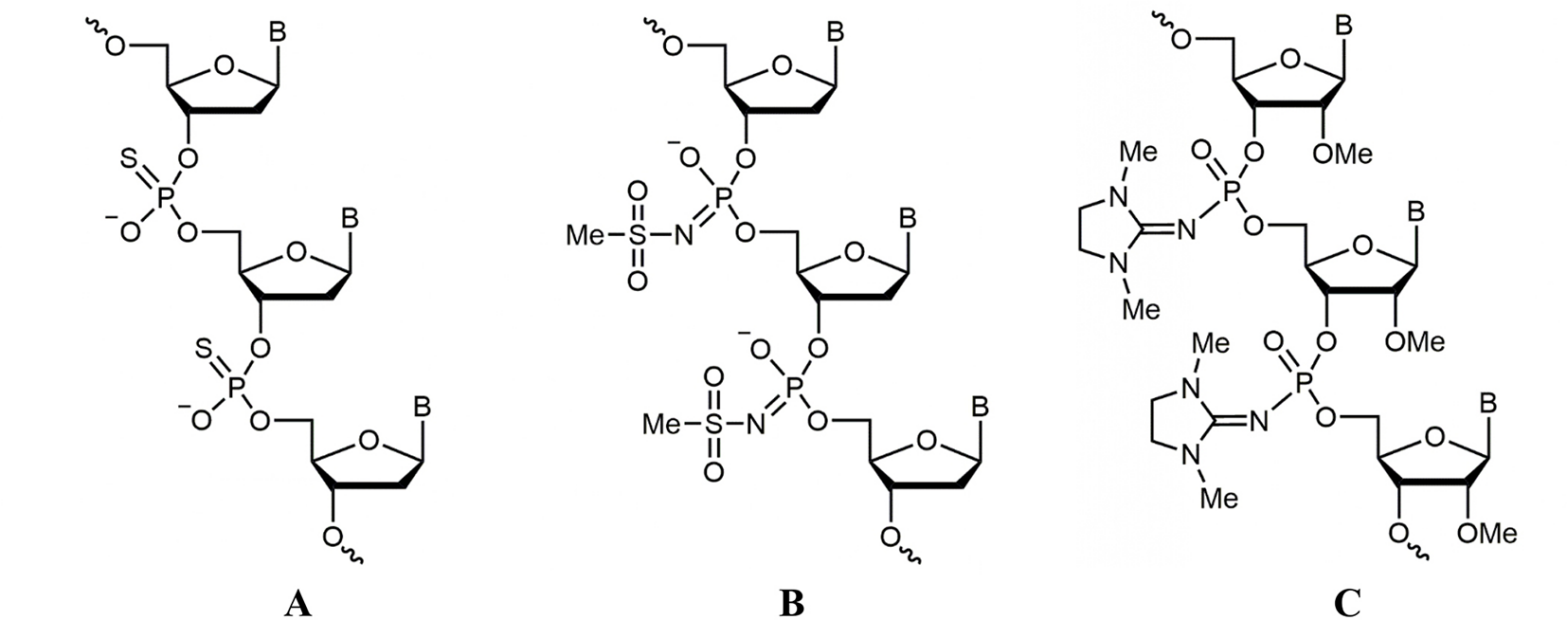
Structures of nucleic acid analogues used in the study. **(A)** Oligodeoxynucleotide phosphorothioate (PS oligodeoxynucleotide (ODN)), **(B)** oligodeoxynucleotide mesyl phosphoramidate (μ-ODN), and **(C)** phosphoryl guanidine oligo-2′-*O*-methylribonucleotide (2′-OMe PGO).

Oligonucleotides used in this study are listed in **Supplementary Table 1**. Oligodeoxynucleotides were synthesized by Eurogen (Russia), and a PS oligodeoxynucleotide (**Figure 1A**) was purchased from DNA Synthesis (Russia). Mesyl phosphoramidate oligodeoxynucleotide μ-*ald* (**Figure 1B**) was synthesized as described previously (Chelobanov et al., 2017), and 2′-OMe PGO-*ald* and FAM-PGO incorporating 1,3-dimethylimidazolidine-2-imino group at each internucleotidic position (**Figure 1C**) were obtained according to the published method (Stetsenko et al., 2014; Su et al., 2019). Synthesis was performed in an automated DNA/RNA synthesizer ASM-800 (Biosset, Russia) using standard 2-cyanoethyl 2′-*O*-methylribonucleoside phosphoramidites (Sigma-Aldrich, USA) and 2′-OMe-rU (Sigma-Aldrich) or 3′-(6-fluorescein) (Glen Research, USA) Controlled Pore Glass supports by substituting Staudinger reaction with 2-azido-1,3-dimethylimidazolinium hexafluorophosphate (TCI, Japan) for iodine oxidation. The reaction was carried out with 0.1 M of 2-azido-1, 3-dimethylimidazolinium hexafluorophosphate in acetonitrile for 15 min at ambient temperature. In case of FAM-PGO, incorporation of the first 2′-OMe-rU residue was followed by iodine oxidation to produce a single phosphodiester linkage, switching to the Staudinger reaction for the rest of the sequence. Oligonucleotides were cleaved from solid support and deprotected by treatment with AMA reagent (25% aq. ammonia and 40% aq. methylamine, 1:1 v/v) at 55°C for 15 min. After the removal of volatiles *in vacuo*, the oligonucleotides were dissolved in 50% acetonitrile and subjected to conventional reversed-phase high-performance liquid chromatography (HPLC) analysis and purification followed by mass spectrometry. Molecular masses of oligonucleotides were determined by electrospray ionization (ESI) liquid chromatography–tandem mass spectrometry (LC-MS/MS) on an Agilent G6410A spectrometer (Agilent Technologies, USA) in a positive ion detection mode using standard device settings. The samples were dissolved in a buffer containing 20 mM of triethylammonium acetate (TEAA) and 60% acetonitrile to 0.1 mM. Sample volume was 10 µL, eluent was 80% aqueous acetonitrile, and flow rate was 0.1 mL/min. Molecular masses of the oligonucleotides were calculated using sets of experimental *m/z* values, which were evaluated for each sample.

### Bacterial Culture

*Mycobacterium smegmatis* mc^2^155 was taken from frozen stocks (bacterial collection of Bach Institute of Biochemistry, Research Center of Biotechnology of the Russian Academy of Sciences, Moscow, Russia) and pre-cultured for 24 h at 37°C on an orbital shaker (200 rpm) in 40 mL of the rich medium Nutrient Broth (Himedia, India) supplemented with 0.05% of Tween-80. To test antibacterial activity of ASOs, the culture was re-grown to fresh medium (same composition) with concentration of approximately 3 × 10^3^ CFU/mL.

### *In Vitro* Growth Inhibition Assay

Antibacterial activity of ASOs against *M. smegmatis* mc^2^155 was examined using colony-forming unit (CFU) counting. Oligonucleotides in concentrations of 10 and 20 µM were added to *M. smegmatis* cells suspensions, and bacteria were cultivated at 37°C, 200 rpm; untreated *M. smegmatis* culture was used as control. Samples were collected at various time points, serially diluted 10-fold, plated on Nutrient Broth agar, and incubated at 37°C for 3 days before counting CFUs. Three independent experiments were performed in four replicates each.

### RNA Isolation

*M. smegmatis* mc^2^155 cultures (10 mL) were centrifuged at 4,000 × *g* for 15 min, and bacterial pellets washed twice with fresh medium, rapidly cooled on ice, and centrifuged again. Total RNA was isolated by phenol-chloroform extraction and cell disruption with Bead Beater (BioSpec Products, USA) as described (Rustad et al., 2009) and treated with Turbo DNase (Life Technologies, USA) to remove traces of genomic DNA.

### cDNA Synthesis and Quantitative (q) Reverse Transcriptase–Polymerase Chain Reaction (RT-PCR)

cDNA was synthesized from 1-mg total RNA using random hexanucleotides and SuperScript III reverse transcriptase (Life Technologies, USA) according to the manufacturer’s protocol. qPCR was performed using qPCRmix-HS SYBR (Evrogen, Russia) in a LightCycler 480 Real-Time PCR system (Roche, Switzerland) at the following cycling conditions: 95°C for 20 s, 61°C for 20 s, and 72°C for 30 s repeated 40 times. Three biological and nine technical replicates were used to ensure reproducibility, and the results were analyzed by LinRegPCR v 2014.6. The results were normalized against 16S rRNA to correct the sample-to-sample variation. Calculations were performed according to Ganger et al. (2017) for the relative expression ratio. PCR primers are listed in **Supplementary Table 1**.

### RNase H Assay

The assay was performed as previously described (Miroshnichenko et al., 2019) with some modifications. Briefly, ASOs (1 µM) were hybridized to complementary RNA (0.1 µM) in 20 mM of Tris–HCl (pH 7.5), 120 mM of KCl, 15 mM of MgCl_2_, and 0.65 mM of EDTA at 68°C to 25°C for 30 min. After annealing, 0.5 U of RNAse H (Thermo Fisher Scientific) was added, and samples were incubated at 37°C for 30 min to allow RNA digestion in RNA : DNA hybrids. The samples were heated with Gel Loading Buffer II (Thermo Fisher Scientific) at 95°C for 5 min and analyzed by denaturing polyacrylamide gel electrophoresis (PAGE) (15% acrylamide, 7 M of urea). RNA and DNA bands were visualized by SYBR Gold staining under UV light.

### Primer Extension Analysis of *Ald* Transcription

Primer 5′-TGACCGCTTCTTCGAGTTCG was labeled at the 5′ end by [γ-^32^P]-ATP using T4 polynucleotide kinase (Thermo Fisher Scientific), and 10 pmol was mixed with 1 μg of total RNA, denatured at 70°C for 10 min, and chilled on ice immediately. Reverse transcription was performed using Superscript III reverse transcriptase (Invitrogen, USA) at 55°C for 1 h and 70°C for 15 min according to the manufacturer’s instructions. The reaction products were mixed with Gel Loading Buffer II, denatured at 90°C for 5 min, and loaded in a 6% acrylamide sequencing gel. After electrophoresis, the gels were subjected to autoradiography on X-ray films (Retina, USA).

### Western Blotting

Bacterial cells were collected and lysed using Bead Beater (BioSpec Products) and heated for 5 min at 95°C in 2× sodium dodecyl sulfate (SDS) sample buffer (100 mM of Tris–HCl, pH 6.8, 4% SDS, 0.2% Bromophenol Blue, 20% glycerol, and 200 mM of DTT). Protein was measured by the Bradford assay. Equal amounts of total protein (5 μg each) were resolved by SDS-PAGE in a 12% gel and transferred onto Hybond-P membranes (GE Healthcare, UK), which were blocked with 5% w/v nonfat dry milk (Bio-Rad, USA), and incubated with primary antibodies against l-alanine dehydrogenase (Ald) (LS-C184923/70850, 1:10,000; LSBio, USA) and then with secondary anti-rabbit horseradish peroxidase (HRP)-conjugated IgG (Cell Signaling Technology, USA). Specific signals were visualized on X-ray films (Retina, USA) using the Immun-Star HRP Chemiluminescent kit (Bio-Rad) and quantified by densitometry using the GelPro software. The specificity of the LS-C184923/70850 antibody to the Ald protein of *M. smegmatis* was additionally confirmed by reaction with recombinant Ald (**Suppl. Materials and Methods**; **Suppl. Figure 1**).

### Cell Viability Assay

The effect of 2′-OMe PGO and ald-PS on cell viability was determined by the MTS assay (CellTiter 96^®^ AQueous One solution Cell Proliferation Assay; Promega, USA). Mouse macrophage RAW 264.7 cells (ATCC TIB-71, a kind gift from Dr. Elena Svirshchevskaya, Shemyakin-Ovchinnikov Institute of Bioorganic Chemistry, Moscow, Russia) were seeded onto 96-well plates at a density of 10,000 cells/well and incubated at 37°C in a humidified atmosphere of 5% CO_2_ overnight. After 24 h, fresh medium containing 10 and 20 μM of oligonucleotides was added for 24 or 48 h; untreated cells were used as controls. Cell survival was assessed by adding 20 μL of MTS reagent into each well for 2 h and measuring the absorbance at 490 nm using a microplate reader (Benchmark Plus Microplate spectrophotometer, Bio-Rad). The assay was done in three biological replicates.

### Confocal Microscopy

The infection procedure was performed as previously described (Bettencourt et al., 2010) with some modifications. RAW 264.7 cells were cultured in RPMI-1640 medium (Gibco Europe, UK) supplemented with 10% fetal bovine serum (FBS, Gibco). The day before infection, cells were seeded at the density of 5 × 10^4^ per well in antibiotic-free medium on cover glasses (18 × 18 mm Menzel Gläsercoverslips, Thermo Fisher Scientific) placed in 6-well culture plates (Costar, USA). *M. smegmatis* cells expressing fluorescent mCherryRed red protein were obtained by electroporation of bacteria with pSMT3-mCherry plasmid (Carroll et al., 2010) and used to infect 80% confluent RAW 264.7 cell monolayers at multiplicity of infection (MOI) 10:1. After 3 h, the medium was removed, cells were washed twice with phosphate-buffered saline (PBS), and fresh medium containing amikacin (100 mg/mL) was added for 3 h to eliminate extracellular mycobacteria. Then, cells were washed twice in PBS and incubated with 10 μM of fluorescein-labeled PGO (FAM-PGO) in RPMI-1640 supplemented with 10% FBS for 3 h. Hoechst 33342 solution (5 µg/mL) was added at the end of incubation for 5 min to stain cell nuclei. After being washed twice with PBS, infected cells were fixed in 1% paraformaldehyde for 10 min and then washed three times with PBS. Mycobacteria were visualized by mCherry fluorescence at 543 nm, FAM-PGO uptake was detected at 488 nm, and cell nuclei were visualized at 405 nm using an Eclipse TE2000 confocal microscope (Nikon, Japan).

### Infection of Macrophages

RAW 264.7 cells were cultured in RPMI-1640 medium (Gibco Europe) supplemented with 10% FBS (Gibco) in 25-cm^2^ culture flasks (Costar) until 70∼80% confluency. *M. smegmatis* cells (OD 0.8, washed PBS) in 2 mL of RPMI-1640 with 10% fetal calf serum (FCS) were added to macrophages at MOI 10:1. After 3 h, the medium was removed, cells were washed twice with PBS, and fresh medium containing amikacin (100 mg/mL) was added for 3 h to eliminate extracellular mycobacteria. Then, cells were washed twice in PBS, and ASOs (*ald*-PGO, scr-PGO) were added to the final concentration of 20 µM. After 4 h, infected macrophage cells were collected, and total RNA was isolated by bacterial cell disruption and phenol-chloroform extraction as described in Section 2.4.

### Statistical Analysis

Statistical analysis was performed using Microsoft office Excel 2007 and GraphPad Prism 6.0 (GraphPad Software Inc., USA). The data were expressed as the mean ± standard deviation. For non-normally distributed data, the Mann–Whitney *U* test was used. Differences were considered statistically significant at *p < 0.05. At least three independent experiments were performed for each assay.

## Results

2′-OMe PGOs are structural derivatives of phosphoryl guanidine oligodeoxynucleotides synthesized by replacing deoxyribose with 2-*O*-methylribose (**Figure 1C**) as described earlier (Kupryushkin et al., 2014). An anti-*ald* 2′-OMe PGO (*ald*-PGO) covering the −10- to +13-nt region of the *ald* mRNA was designed to overlap the Shine-Dalgarno sequence and include the start codon. To elucidate the effects of antisense 2′-OMe PGs as anti-mycobacterial agents, we used non-pathogenic fast-growing *M. smegmatis*, which shares about 79% nucleotide sequence homology with *M. tuberculosis*, is very similar to it in cell wall composition and metabolic processes (Chaturvedi et al., 2007), and is widely used as a laboratory model of pathogenic *M. tuberculosis*. The *ald* gene (MSMEG_2659) encoding alanine dehydrogenase, which may play a role in cell wall synthesis as l-alanine is an important constituent of the peptidoglycan layer (Dave and Kadeppagari, 2019), was selected as a target because its downregulation is not fatal for mycobacteria.

### Ald-PGO Slows *in Vitro* Growth of *M. smegmatis*

**Figure 2:**
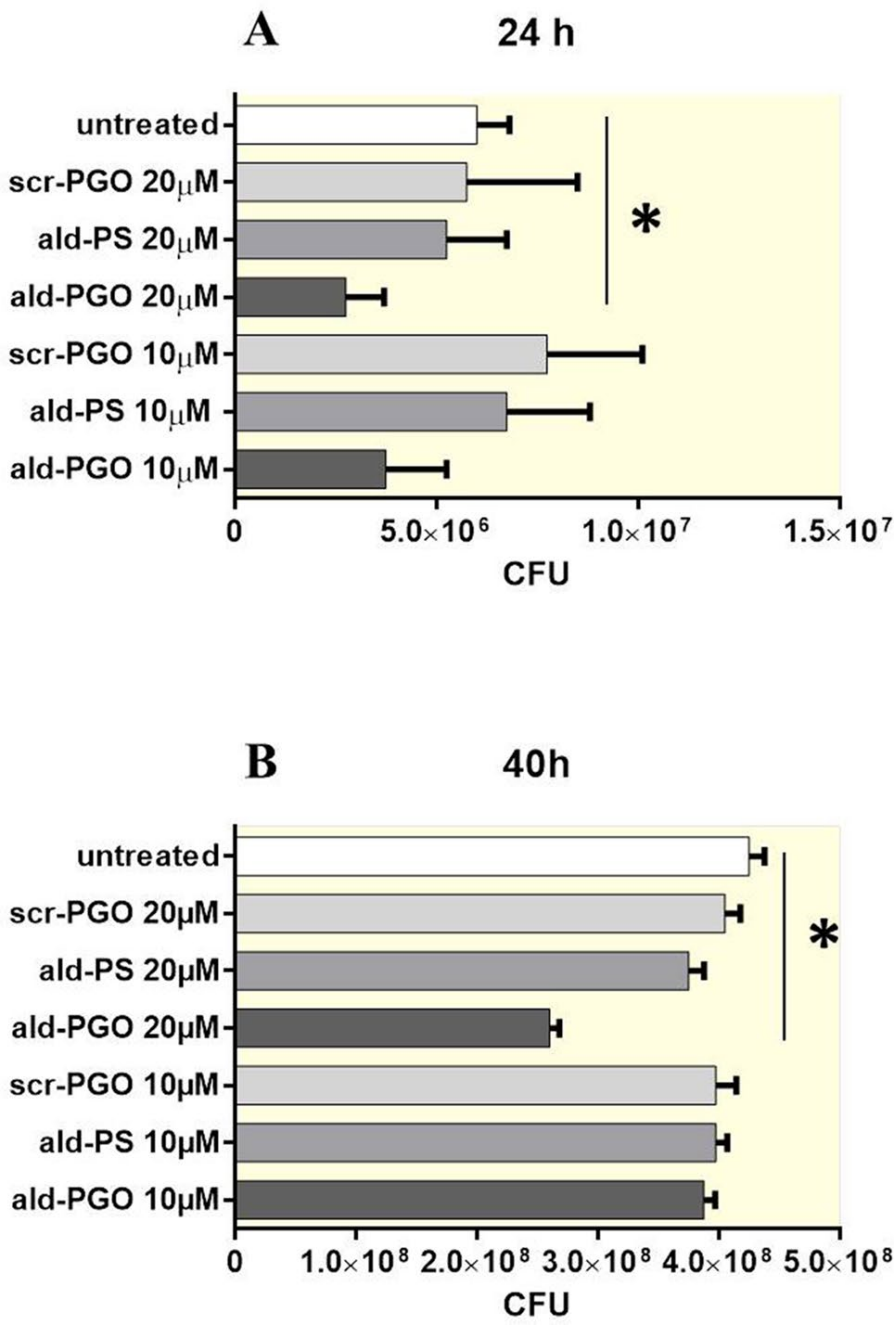
Growth of *Mycobacterium smegmatis* cultures in the presence of different ASOs at different time points after ASO treatment: 24 h **(A)** and 40 h **(B)**. Mycobacteria were treated with 10 and 20 μM of *ald*-PGO, *ald*-PS, and scr-PGO, or left untreated for the indicated times. The data are presented as the mean ± SD of three independent experiments; *p < 0.05. ASOs, antisense oligonucleotides.

The bacteria were treated with two concentrations of oligonucleotides *ald*-PGO, *ald*-PS, and a scrambled 2′-OMe PGO (scr-PGO) used as control, and the CFUs were counted at 24 and 40 h after treatment. The results indicated that *ald*-PGO was more efficient in suppressing *M. smegmatis* growth than *ald*-PS. At both concentrations used, *ald*-PGO inhibited the growth of *M. smegmatis*, and the effect was statistically significant at 20 μM (54% inhibition compared with untreated control at 24 h, 62% at 40 h), whereas *ald*-PS and scr-PGO did not slow bacterial growth (**Figure 2**). After 48 h, the growth rates of all cultures were approaching those of the control (data not shown), probably because a single addition of *ald*-PGO to the culture was not sufficient to induce prolonged effects.

### *Ald*-PGO Downregulates Ald Expression at the Translational Level

**Figure 3:**
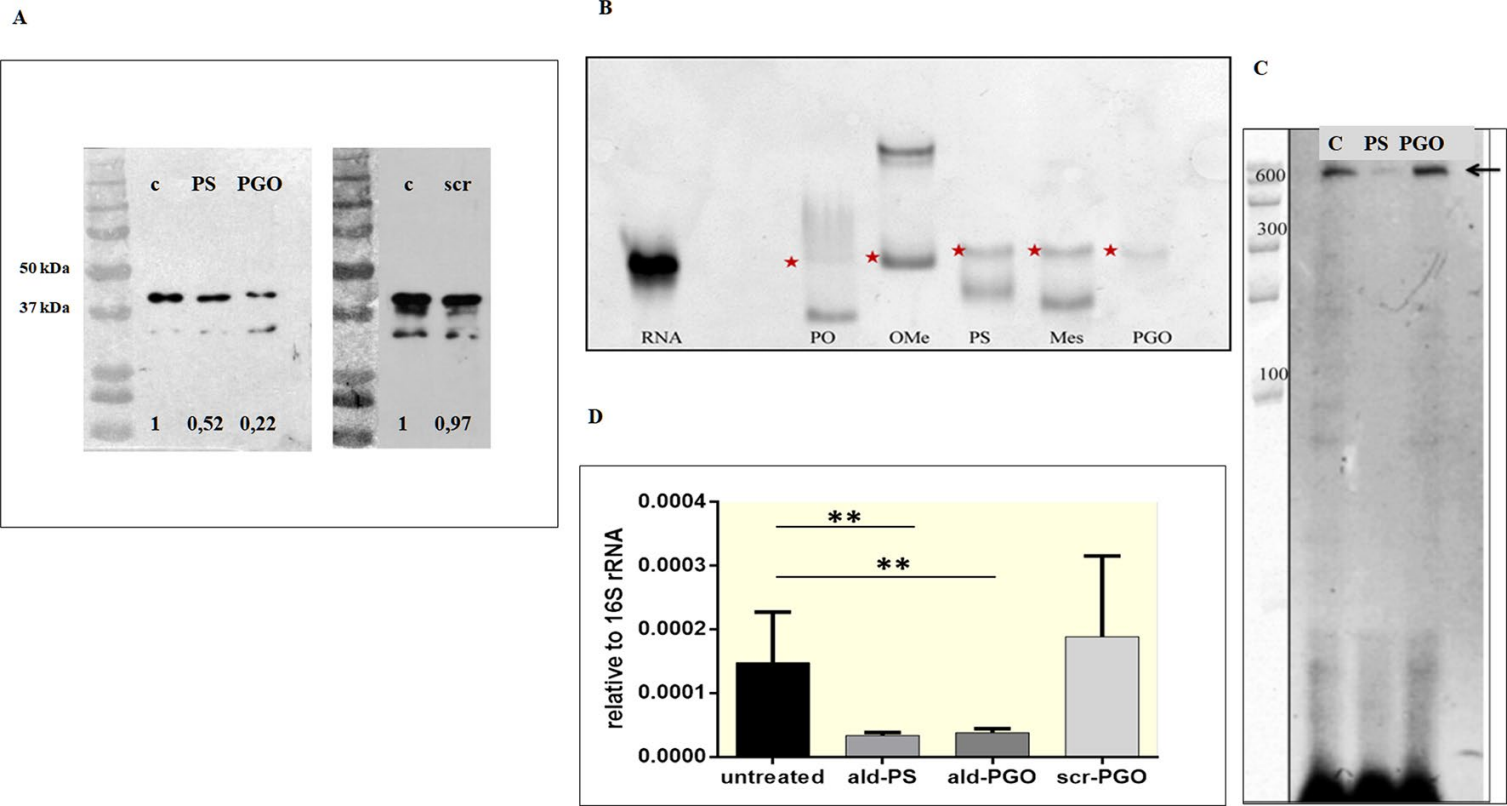
2′-OMe PGO inhibits the expression of the target gene in *Mycobacterium smegmatis*. **(A)** Specific inhibition of protein synthesis by antisense *ald*-PGO. *M. smegmatis* cultures were treated with 20 μM of *ald*-PGO (PGO), *ald*-PS (PS), and scr-PGO (scr) or left untreated (control, C) for 24 h and analyzed for Ald protein expression by western blotting; numbers below indicate the ratio to control. **(B)** Cleavage of ASO : RNA duplexes by RNase H. Target RNA duplexes with *ald* (PO), *ald*-oligo-2′-*O*-methylribonucleotide (OMe), *ald*-PS (PS), μ-*ald* (Mes), and *ald*-PGO (PGO) were treated by RNAse H and analyzed by PAGE in a denaturing 15% gel, which was stained with SYBR Green. Red asterisks indicate RNA. **(C)** Primer extension analysis of the *ald* gene transcription. RNA was isolated from *M. smegmatis* cultures treated with 20 μM of *ald*-PGO (PGO) and *ald*-PS (PS) or left untreated (control, C) for 24 h and reverse transcribed using a radioactively labeled *ald*-specific oligonucleotide. The arrow indicates the transcription start point. RiboRuler RNA ladder (Thermo Fisher Scientific) is shown at the left. **(D)** Quantification of *ald* transcription. *M. smegmatis* cultures were treated with 20 μM of the indicated ASOs for 24 h and analyzed for *ald* mRNA expression by qRT-PCR. The data are normalized to the 16S rRNA transcription level. **p < 0.01. 2′-OMe PGO, phosphoryl guanidine oligo-2′-*O*-methylribonucleotide; PAGE, polyacrylamide gel electrophoresis; qRT-PCR, quantitative reverse transcriptase–polymerase chain reaction

To determine whether the decrease of *M. smegmatis* growth by 2′-OMe PGO was related to specific inhibition of Ald synthesis, we assessed Ald protein expression in ASO-treated cultures by western blotting. The samples were equalized for total protein amount by the Bradford method, and then evaluated by densitometry (**Supplementary Figure 2**). The *ald*-PGO-treated bacteria showed a much more significant reduction (22% of the untreated control) of Ald expression than PS-treated cells (52%), whereas no apparent difference in Ald protein levels was observed for scr-PGO-treated cells compared with control (**Figure 3A**). These results suggest that the suppression of *M. smegmatis* growth by *ald*-PGO was associated with its specific antisense activity to inhibit Ald expression.

### 2′-OMe PGO Did Not Activate RNase H

ASOs can inhibit gene expression by steric blocking or through degradation of the RNA strand in a hybrid duplex by bacterial RNases. To determine the mechanism underlying the downregulation of gene expression by 2′-OMe PGO, we evaluated the sensitivity of ASO-complementary RNA hybrids to RNase H-mediated degradation. An unmodified oligodeoxyribonucleotide (*ald*), *ald*-PS, and mesyl phosphoramidate oligodeoxynucleotide (μ-*ald*) were used as positive controls, as these structure were shown to activate RNase H (Miroshnichenko et al., 2019), whereas oligo-2′-*O*-methylribonucleotide (*ald*-OMe), which forms an RNase H-resistant duplex, was used as a negative control. The oligonucleotides were hybridized with *ald* RNA and treated with RNase H. As expected, there was a decrease in *ald* RNA band intensity for duplexes with *ald*, *ald*-PS, and µ-*ald*, indicating cleavage by RNase H, whereas no traces of digested *ald*-RNA were observed for the duplex with *ald*-PGO (**Figure 3B**). The PGO-RNA duplex did not penetrate the gel at pH 8.3 because of its neutral charge (Fokina et al., 2018); the visible traces of *ald*-RNA could be attributed to the possible presence of non-hybridized RNA. These results indicate that duplexes of 2′-OMe PGO with the target RNA are stable even under denaturing conditions and that 2′-OMe PGO does not activate RNAse H.

### Target mRNA Remained Intact After 2′-OMe PGO Treatment of *M. smegmatis*

To validate the results of direct molecular interactions on the cellular level, we assessed the presence of intact *ald* mRNA in ASO-treated and control samples by the primer extension method. RNA was isolated from mycobacterial cultures exposed to 20 μM of *ald*-PGO and *ald*-PS and reverse transcribed using a radioactively labeled *ald*-specific oligonucleotide. The identified transcriptional start site coincided with the predicted one at position −27-bp upstream of the ATG start codon (Feng et al., 2002). Furthermore, it was found that full-size *ald* mRNA was significantly decreased in mycobacteria incubated with *ald*-PS, but remained intact in those incubated with *ald*-PGO (**Figure 3C**). These results confirm that *ald*-PGO does not activate RNase H.

### 2′-OMe PGO Treatment of *M. smegmatis* Decreases the Transcription of Its mRNA Target

**Figure 4:**
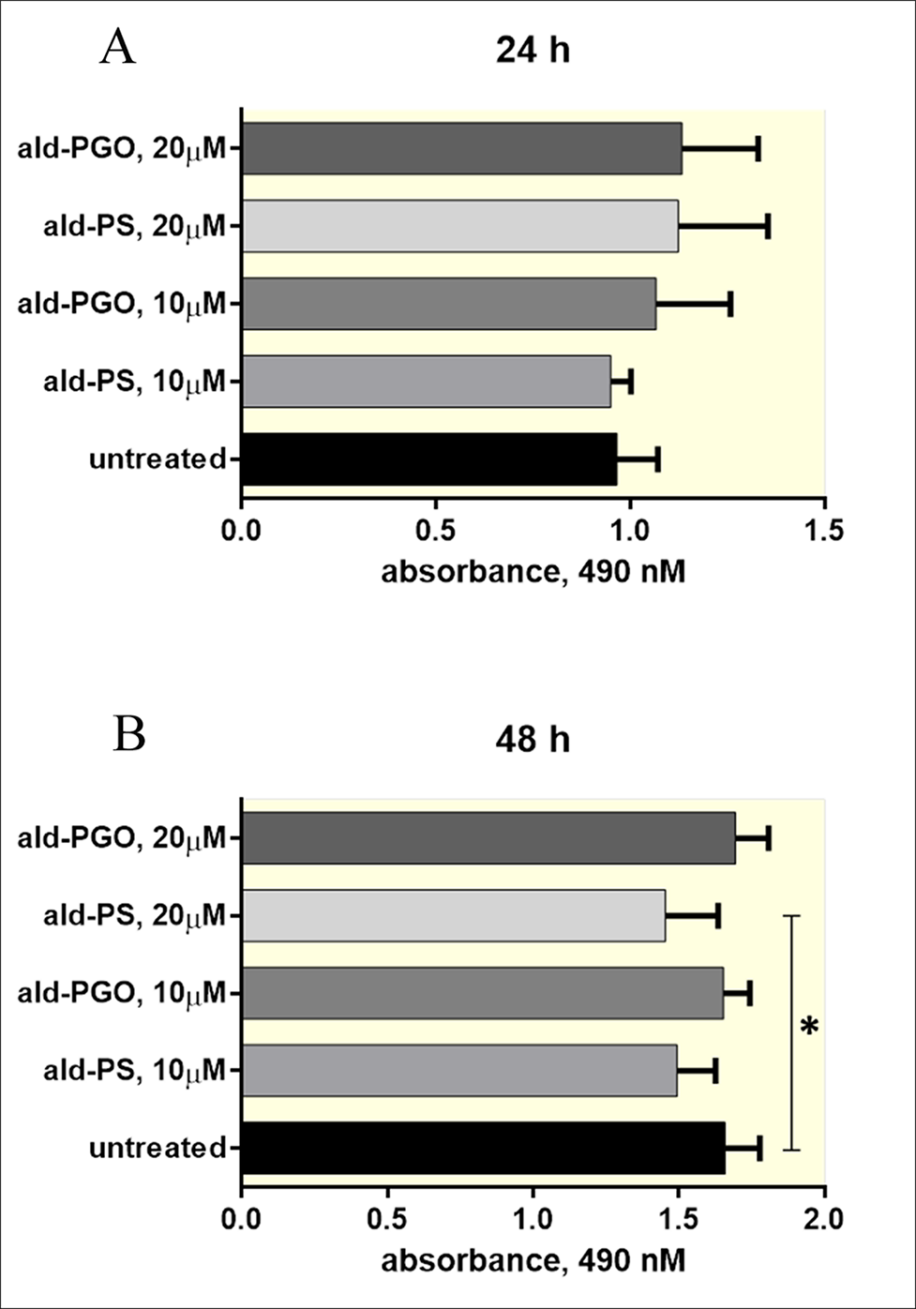
2′-OMe PGO is not toxic for macrophages. RAW 264.7 cells were treated with 10 and 20 μM of *ald*-PGO (PGO) or *ald*-PS (PS) for 24 **(A)** and 48 h **(B)** and analyzed for viability by the MTS assay. *p < 0.05. 2′-OMe PGO, phosphoryl guanidine oligo-2′-*O*-methylribonucleotide.

To quantitatively evaluate *ald* transcription in mycobacteria exposed to ASOs, we performed qRT-PCR. Both *ald*-PS and *ald*-PGO at 20 μM decreased *ald* mRNA expression in *M. smegmatis* after 24-h treatment than did scr-PGO and control cultures (**Figure 3D**). Considering that *ald*-PGO did not activate RNAse H and prevented *ald* mRNA degradation in the duplex (**Figure 4**), these results suggest that 2′-OMe PGO may act as a steric blocker slowing transcription of the target mRNA.

### 2′-OMe PGO Is Not Toxic to Macrophages

As antisense 2′-OMe PGOs are supposed to be used against intracellular bacteria, we tested cytotoxicity of *ald*-PGO for macrophages, in which mycobacteria reside and manage to survive. The results indicated that *ald*-PGO in concentrations 10 and 20 μM did not affect the viability of RAW 264.7 macrophages after 24- or 48-h incubation (**Figure 4**). However, *ald*-PS at 20 μM caused a significant decrease in cell proliferation at 48 h than did control (*p* < 0.05).

### 2′-OMe PGO Is Delivered by Gymnosis Into Mycobacteria Residing in Macrophages and Targets Intracellular Mycobacteria

**Figure 5:**
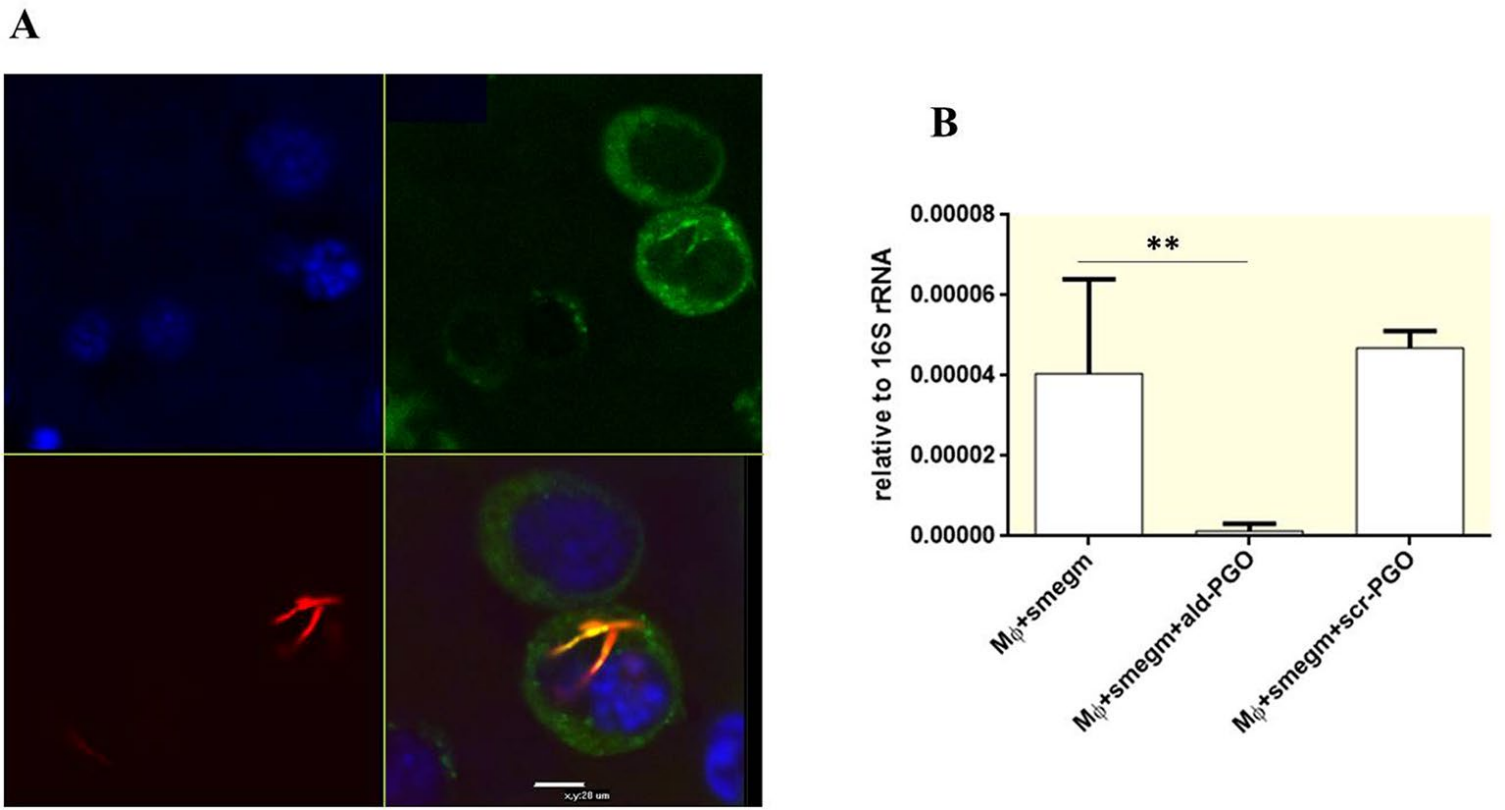
Intracellular effect of 2′-OMe PGO on mycobacteria. **(A)** Confocal microscopy of RAW 264.7 macrophages infected with *Mycobacterium smegmatis* expressing mCherry Red fluorescent protein. The infected cells were treated with fluorescently labeled *ald*-PGO (FAM-PGO, 10 μM) for 24 h and analyzed by confocal microscopy. *M. smegmatis* is red, FAM-PGO is green, and nuclei stained with Hoecst33342 are blue. **(B)** Quantification of *ald* transcription in intracellular *M. smegmatis*. RAW 264.7 cells were infected with mycobacteria, treated with 20 μM of the indicated ASOs, and analyzed for *ald* mRNA expression by qRT-PCR. The data are normalized to the 16S rRNA transcription level. **p < 0.01. 2′-OMe PGO, phosphoryl guanidine oligo-2′-*O*-methylribonucleotide; ASOs, antisense oligonucleotides; qRT-PCR, quantitative reverse transcriptase–polymerase chain reaction.

To determine whether 2′-OMe PGO could penetrate the cell wall of intracellular mycobacteria, RAW 264.7 macrophages were infected by *M. smegmatis* expressing red fluorescent protein, treated by fluorescein-labeled *ald*-PGO (FAM-PGO), and analyzed for FAM-PGO uptake by laser confocal microscopy. The images of infected RAW 264.7 macrophages incubated with FAM-PGO (10 μM) for 6 h revealed that the FAM-PGO was abundantly present in the cytosol and, most importantly, was also taken up by mycobacteria, as evidenced by colocalization of FAM-PGO with fluorescent mCherry protein labeling bacterial cells (**Figure 5A**). These results suggest that 2′-OMe PGO could be delivered to mycobacteria residing in macrophages in the absence of any transfection agent, that is, by gymnosis. To determine whether *ald*-PGO treatment influences the transcription of the target *ald* gene in *M. smegmatis*-infected macrophages, we performed quantitative RT-PCR analysis using total RNA isolated from infected macrophages. qPCR revealed a significant decrease of *ald* transcripts after *ald*-PGO treatment compared with untreated control, or scr-PGO treatment, indicating the intracellular effect of 2′-OMe PGO on mycobacteria (**Figure 5B**).

## Discussion

In this study, we provided evidence that an antisense 2′-OMe PGO could inhibit growth of mycobacteria, downregulate the expression of bacterial genes, and penetrate into intracellular mycobacteria through gymnosis. The 2′-OMe PGO specific for the *ald* gene of *M. smegmatis* effectively reduced the expression of the Ald protein (**Figure 3A**), showing a stronger effect than the *ald*-PS ASO, despite its apparent inability to activate RNase H (**Figure 3B**). It is interesting that, while the target mRNA in the hybrid with PGO remains intact as evidenced by primer extension experiments, its expression is obviously decreased as shown by qRT-PCR analysis (**Figures 3C, D**). This effect needs further investigation, but it can be hypothesized that PGO strongly bound to its target RNA may somehow hamper its transcription through steric blocking. Most importantly, 2′-OMe PGO could enter *M. smegmatis* cells residing inside mouse macrophages without damaging the host cells and downregulate expression of a target mycobacterial gene (**Figures 4** and **5**). To the best of our knowledge, this is the first example of an ASO derivative passing successfully through two cellular barriers, the outer membrane of the eukaryotic cell and the bacterial cell wall. Cumulatively, these findings suggest that PGOs may become a potential platform for designing efficient and specific drugs against mycobacteria.

Nucleic acid derivatives have been long considered as promising candidate therapeutic agents because of their unique ability to selectively bind complementary sequences of target mRNA and downregulate gene expression through the antisense mechanism (Uhlmann and Peyman, 1990; Kurreck, 2003). Extensive synthetic chemistry research has produced a number of structural approaches to improve ASO target affinity, biological stability, pharmacokinetic properties, and safety, resulting in PS oligonucleotides, morpholinos, PNAs, and many others (Shen and Corey, 2018). Several oligonucleotide-based therapies have been approved for clinical application (Stein and Castanotto, 2017).

However, in general, oligonucleotides have poor cellular uptake, which is due to their physicochemical properties such as high hydrophilicity and large net negative charge, and mostly cross the cell membrane barrier through various mechanisms of receptor-mediated endocytosis, which have low efficiency (Juliano, 2016). To ensure a desired therapeutic effect, it is often necessary to use special delivery methods, which may be complicated and increase the cost of treatment. Therefore, ASOs that could be delivered by gymnosis, that is, without the aid of any transfection reagents, are currently under development.

To date, there are no examples of successful clinical use of ASOs as antibacterial agents. The emergence of MDR and XDR *M. tuberculosis* strains in recent years requires new more effective therapeutic strategies, which would not promote drug resistance of the pathogen (Parida et al., 2015). In their pioneering work, Harth et al. (2000) used synthetic PS oligonucleotide derivatives against *M. tuberculosis*, demonstrating a fundamental proof of principle by showing that PS ASOs inhibited synthesis of glutamine synthase I (GlnA1) and its activity and suppressed bacterial growth. A more pronounced effect was achieved by using a combination of three different PS ASOs targeting different parts of the *glnA1* gene or inhibiting three mycolyltransferase genes, *fbp A*, *B*, and *C* (Harth et al., 2002; Harth et al., 2007). However, this work was not continued, possibly because of insufficient affinity of PS ASOs to structured RNAs and ineffective penetration through the cell wall of mycobacteria.

More success was gained using PNAs, another class of ASOs conjugated with peptides. Kulyte et al. (2005) demonstrated that a PNA oligomer targeting the *inhA* gene of *M. smegmatis* caused a significant retardation of bacterial growth and induced changes in cellular morphology typical for InhA mutants. The potential of antisense antimicrobials has been demonstrated by studies on peptide conjugates of PMOs, which showed significant inhibitory effects against MDR strains of *Burkholderia multivorans* and *Acinetobacter baumannii* both *in vitro* and *in vivo* (Sully and Geller, 2016; Daly et al., 2017) as well as against intracellular *Toxoplasma gondii* (Lai et al., 2012). However, their activity against mycobacteria is unknown.

A new type of RNA analogues 2′-OMe PGOs (**Figure 1C**) tested in this study have been shown to be electrostatically neutral, possess high resistance to cellular nucleases, and form stable duplexes with target RNA targets (Stetsenko et al., 2014). In the present study, investigation of the biological activity of 2′-OMe PGOs revealed that they exerted stronger inhibitory effects on mycobacteria, had higher affinity to the target mRNA than PS ASOs, and are taken up by intracellular mycobacteria.

Thus, our findings indicate that novel 2′-OMe PGOs have a potential as antisense antimicrobial drugs, which may find application in the future as promising therapeutic agents against TB, especially its drug-resistant forms.

## Data Availability

The raw data supporting the conclusions of this manuscript will be made available by the authors, without undue reservation, to any qualified researcher.

## Author Contributions

DS designed the 2′-OMe PGO RNA analogues. TA and DS conceived and designed the experiments. EB carried out oligonucleotide syntheses and analyses. YS, ES, and OB performed biological experiments. TA and DS analyzed the data. TA, ES, YS, and DS prepared figures and graphs. TA and DS wrote the paper. All the authors have read and approved the final manuscript.

## Funding

This work was funded by RSF grants 15-15-00121 (to DS) and 18-15-00332 (to TA, red fluorescent *M. smegmatis* strain preparation, infection, and confocal microscopy experiments). EB and DS were partially supported by the Russian government-funded budget project VI.62.1.3, 0309-2016-0005 (2017-2020) “Therapeutic nucleic acids.”

## Conflict of Interest Statement

The authors declare that the research was conducted in the absence of any commercial or financial relationships that could be construed as a potential conflict of interest.

## References

[B1] AlMatar M. AlMandeal H. Var I. Kayar B. Koksal F. (2017). New drugs for the treatment of Mycobacterium tuberculosis infection. Biomed. Pharmacother. 91, 546–558. 10.1016/j.biopha.2017.04.105 28482292

[B2] Altman S. (2014). Antibiotics present and future. FEBS Letters 588, 1–2. 10.1016/j.febslet.2013.10.048 24252220

[B3] Bettencourt P. Pires D. Carmo N. Anes E. (2010). “Application of confocal microscopy for quantification of intracellular mycobacteria in macrophages,” in Microscopy: Science, technology, applications and education, ed. Díaz A. M. (Badajoz, Spain: Formatex Research Center), 614–621.

[B4] Carroll P. Schreuder L. J. Muwanguzi-Karugaba J. Wiles S. Robertson B. D. Ripoll J. (2010). Sensitive detection of gene expression in mycobacteria under replicating and non-replicating conditions using optimized far-red reporters. PLoS One 5, e9823. 10.1371/journal.pone.0009823 20352111 PMC2843721

[B5] Chaturvedi V. Dwivedi N. Tripathi R. P. Sinha S. (2007). Evaluation of Mycobacterium smegmatis as a possible surrogate screen for selecting molecules active against multi-drug resistant Mycobacterium tuberculosis. J. Gen. Appl. Microbiol. 53, 333–337. 10.2323/jgam.53.333 18187888

[B6] Chelobanov B. Burakova E. Prokhorova D. Fokina A. Stetsenko D. (2017). New oligodeoxynucleotide derivatives containing N-(methanesulfonyl)-phosphoramidate (mesyl phosphoramidate) internucleotide group. Russ. J. Bioorg. Chem. 43, 664–668. 10.1134/S1068162017060024

[B7] Daly S. M. Sturge C. R. Greenberg D. E. (2017). “Inhibition of bacterial growth by peptide-conjugated morpholino oligomers,” in Morpholino Oligomers, eds. Moulton H. Moulton J. (New York, NY: Humana Press), 115–122. 10.1007/978-1-4939-6817-6_10. PMC5497461 28364238

[B8] Dave U. C. Kadeppagari R. K. (2019). Alanine dehydrogenase and its applications—a review. Crit. Rev. Biotechnol. 39, 648–664. 10.1080/07388551.2019.1594153 31018703

[B9] Dowdy S. F. (2017). Overcoming cellular barriers for RNA therapeutics. Nat. Biotechnol. 35, 222–229. 10.1038/nbt.3802 28244992

[B10] Feng Z. Caceres N. E. Sarath G. Barletta R. G. (2002). Mycobacterium smegmatis l-alanine dehydrogenase (Ald) is required for proficient utilization of alanine as a sole nitrogen source and sustained anaerobic growth. J. Bacteriol. 184, 5001–5010. 10.1128/JB.184.18.5001-5010.2002 12193615 PMC135311

[B11] Fokina A. Wang M. Ilyina A. Klabenkova K. Burakova E. Chelobanov B. (2018). Analysis of new charge-neutral DNA/RNA analogues phosphoryl guanidine oligonucleotides (PGO) by gel electrophoresis. Anal. Biochem. 555, 9–11. 10.1016/j.ab.2018.05.027 29864402

[B12] Fokina A. A. Chelobanov B. P. Fujii M. Stetsenko D. A. (2017). Delivery of therapeutic RNA-cleaving oligodeoxyribonucleotides (deoxyribozymes): from cell culture studies to clinical trials. Expert Opin. Drug Delivery 14, 1077–1089. 10.1080/17425247.2017.1266326 27892730

[B13] Ganger M. T. Dietz G. D. Ewing S. J. (2017). A common base method for analysis of qPCR data and the application of simple blocking in qPCR experiments. BMC Bioinf. 18, 534. 10.1186/s12859-017-1949-5 PMC5709943 29191175

[B14] Good L. Awasthi S. K. Dryselius R. Larsson O. Nielsen P. E. (2001). Bactericidal antisense effects of peptide–PNA conjugates. Nat. Biotechnol. 19, 360. 10.1038/86753 11283595

[B15] Harth G. Zamecnik P.C. Tang J.Y. Tabatadze D. Horwitz M.A. (2000). Treatment of Mycobacterium tuberculosis with antisense oligonucleotides to glutamine synthetase mRNA inhibits glutamine synthetase activity, formation of the poly-L-glutamate/glutamine cell wall structure, and bacterial replication. Proc. Natl. Acad. Sci. U. S. A. 97, 418–423. 10.1073/pnas.97.1.418 10618433 PMC26678

[B16] Harth G. Horwitz M. A. Tabatadze D. Zamecnik P. C. (2002). Targeting the Mycobacterium tuberculosis 30/32-kDa mycolyl transferase complex as a therapeutic strategy against tuberculosis: proof of principle by using antisense technology. Proc. Natl. Acad. Sci. U. S. A. 99, 15614–15619. 10.1073/pnas.242612299 12427974 PMC137765

[B17] Harth G. Zamecnik P. C. Tabatadze D. Pierson K. Horwitz M. A. (2007). Hairpin extensions enhance the efficacy of mycolyl transferase-specific antisense oligonucleotides targeting Mycobacterium tuberculosis. Proc. Natl. Acad. Sci. U. S. A. 104, 7199–7204. 10.1073/pnas.0701725104 17438292 PMC1855390

[B18] Hegarty J. P. Stewart D.B. (2018). Advances in therapeutic bacterial antisense biotechnology. Appl. Microbiol. Biotechnol. 102, 1055–1065. 10.1007/s00253-017-8671-0 29209794 PMC5777866

[B19] Juliano R. L. (2016). The delivery of therapeutic oligonucleotides. Nucleic Acids Res. 44, 6518–6548. 10.1093/nar/gkw236 27084936 PMC5001581

[B20] Kulyte A. Nekhotiaeva N. Awasthi S. K. Good L. (2005). Inhibition of Mycobacterium smegmatis gene expression and growth using antisense peptide nucleic acids. J. Mol. Microbiol. Biotechnol. 9, 101–109. 10.1159/000088840 16319499

[B21] Kupryushkin M. S. Pyshnyi D. V. Stetsenko D. A. (2014). Phosphoryl guanidines: a new type of nucleic acid analogues. Acta Naturae 6, 116–118. 10.32607/20758251-2014-6-4-116-118 25558402 PMC4273099

[B22] Kurreck J. (2003). Antisense technologies: improvement through novel chemical modifications. Eur. J. Biochem. 270, 1628–1644. 10.1046/j.1432-1033.2003.03555.x 12694176

[B23] Lai B. S. Witola W. H. El Bissati K. Zhou Y. Mui E. Fomovska A. (2012). Molecular target validation, antimicrobial delivery, and potential treatment of Toxoplasma gondii infections. Proc. Natl. Acad. Sci. U. S. A. 109, 14182–14187. 10.1073/pnas.1208775109 22891343 PMC3435209

[B24] Lomzov A. A. K. Shernyukov A. V. Nekrasov M.D. Dovydenko I. S. Stetsenko D. A. Pyshnyi D. V. (2019). Diastereomers of a mono-substituted phosphoryl guanidine trideoxyribonucleotide: isolation and properties. Biophys. Biochem. Res. Commun. 14, 1212–1220. 10.1016/j.bbrc.2019.04.024 31000201

[B25] Miroshnichenko S. Patutina O. Burakova E. Chelobanov B. Fokina A. Vlassov V. (2019). Mesyl phosphoramidate antisense oligonucleotides as an alternative to phosphorothioates with improved biochemical and biological properties. Proc. Natl. Acad. Sci. 116, 1229–1234. 10.1073/pnas.1813376116 30622178 PMC6347720

[B26] Parida S. K. Axelsson-Robertson R. Rao M. V. Singh N. Master I. Lutckii A. (2015). Totally drug-resistant tuberculosis and adjunct therapies. J. Intern Med. 277, 388–405. 10.1111/joim.12264 24809736

[B27] Penchovsky R. Traykovska M. (2015). Designing drugs that overcome antibacterial resistance: where do we stand and what should we do? Expert Opin. Drug Discovery 10, 631–650. 10.1517/17460441.2015.1048219 25981754

[B28] Rustad T. R. Roberts D. M. Liao R. P. Sherman D. R. (2009). Isolation of mycobacterial RNA. Methods Mol. Biol. 465, 13–21. 10.1007/978-1-59745-207-6_2 20560069

[B29] Shen X. Corey D. R. (2018). Chemistry, mechanism and clinical status of antisense oligonucleotides and duplex RNAs. Nucleic Acids Res. 46, 1584–1600. 10.1093/nar/gkx1239 29240946 PMC5829639

[B30] Stein C. A. Castanotto D. (2017). FDA-approved oligonucleotide therapies in 2017. Mol. Ther. 25, 1069–1075. 10.1016/j.ymthe.2017.03.023 28366767 PMC5417833

[B31] Stetsenko D.A. Kupryushkin M. S. Pyshnyi D. V. (2014) International patent application WO2016028187A1

[B32] Su Y. Fujii H. Burakova E. A. Chelobanov B. P. Fujii M. Stetsenko D. A. (2019). Neutral and negatively charged phosphate modifications altering thermal stability, kinetics of formation and monovalent ion dependence of DNA G-quadruplexes. Chem. Asian J. 10.1002/asia.201801757 30600926

[B33] Sully E. K. Geller B. L. (2016). Antisense antimicrobial therapeutics. Curr. Opin. Microbiol. 33, 47–55. 10.1016/j.mib.2016.05.017 27375107 PMC5069135

[B34] Uhlmann E. Peyman A. (1990). Antisense oligonucleotides: a new therapeutic principle. Chem. Rev. 90, 543–584. 10.1021/cr00102a001

[B35] Veatch A. V. Kaushal D. (2018). Opening pandora’s box: mechanisms of Mycobacterium tuberculosis resuscitation. Trends Microbiol. 26, 145–157. 10.1016/j.tim.2017.08.001 28911979 PMC5794633

[B36] Xue X. Y. Mao X. G. Zhou Y. Chen Z. Hu Y. Hou Z. (2018). Advances in the delivery of antisense oligonucleotides for combating bacterial infectious diseases. Nanomedicine 14, 745–758. 10.1016/j.nano.2017.12.026 29341934

[B37] Zamecnik P. C. Stephenson M. L. (1978). Inhibition of Rous sarcoma virus replication and cell transformation by a specific oligodeoxynucleotide. Proc. Natl. Acad. Sci. U. S. A. 75, 280–284 10.1073/pnas.75.1.280. 75545 PMC411230

