# Supplementary material for: A New Antisense Phosphoryl Guanidine Oligo-2′-O-Methylribonucleotide Penetrates Into Intracellular Mycobacteria and Suppresses Target Gene Expression
Source: Front Pharmacol. 2019 Sep 19;10:1049. doi: 10.3389/fphar.2019.01049 (PMC6778816; doi:10.3389/fphar.2019.01049)
Supplement: Supplementary file 1 [file DataSheet_1.pdf]

Supplementary Materials and Methods

Specificity of the antibody against *M. smegmatis* L-alanine dehydrogenase (LS-C184923/70850, LSBio) was additionally confirmed using recombinant the Ald protein. The *ald* gene (MSMEG 2659) was amplified with primers *ald_Bam*HI-for 5′-AGGATCCATGCTCGTCGGAATCCCGACC-3′ and *ald_Eco*RI-rev 5′-AGAATTCGAACGGCAGGTCCAGATCCTTG-3′, and cloned into the expression vector pGEX-4T-1 (# 28-9545-49, GE Healthcare, USA) into *Bam*HI and *Eco*RI restriction sites, yielding a fragment fused with the glutathione S-transferase gene. The recombinant plasmid was used to transform competent *E. coli* BL21(DE3) cells (Evrogen, Russia) and expression of the fusion GST-Ald protein was inducted by 0,1 mM isopropyl β-D-1-thiogalactopyranoside (IPTG), according to the manufacturer's instructions (GE Healthcare). Bacterial cells were lysed, total protein measured by the Bradford assay, and equal amounts of protein were resolved in a 12% SDS-PAGE gel and transferred onto a Hybond-P membrane (GE Healthcare, USA). The membrane was blocked with nonfat dry milk (Bio-Rad), and incubated with primary mouse antibodies against GST (1:1,000; B-14 clone, #sc-138, Santa Cruz Biotechnology, USA) and rabbit antibodies against L-alanine dehydrogenase (1:20,000; LS-C184923/70850, LSBio), followed by incubation with secondary anti-mouse or anti-rabbit, respectively, HRP-conjugated IgG (1:2,000; Cell Signaling Technology, USA).
