# Supplementary figures and images for: A New Antisense Phosphoryl Guanidine Oligo-2′-O-Methylribonucleotide Penetrates Into Intracellular Mycobacteria and Suppresses Target Gene Expression
Source: Front Pharmacol. 2019 Sep 19;10:1049. doi: 10.3389/fphar.2019.01049 (PMC6778816; doi:10.3389/fphar.2019.01049)

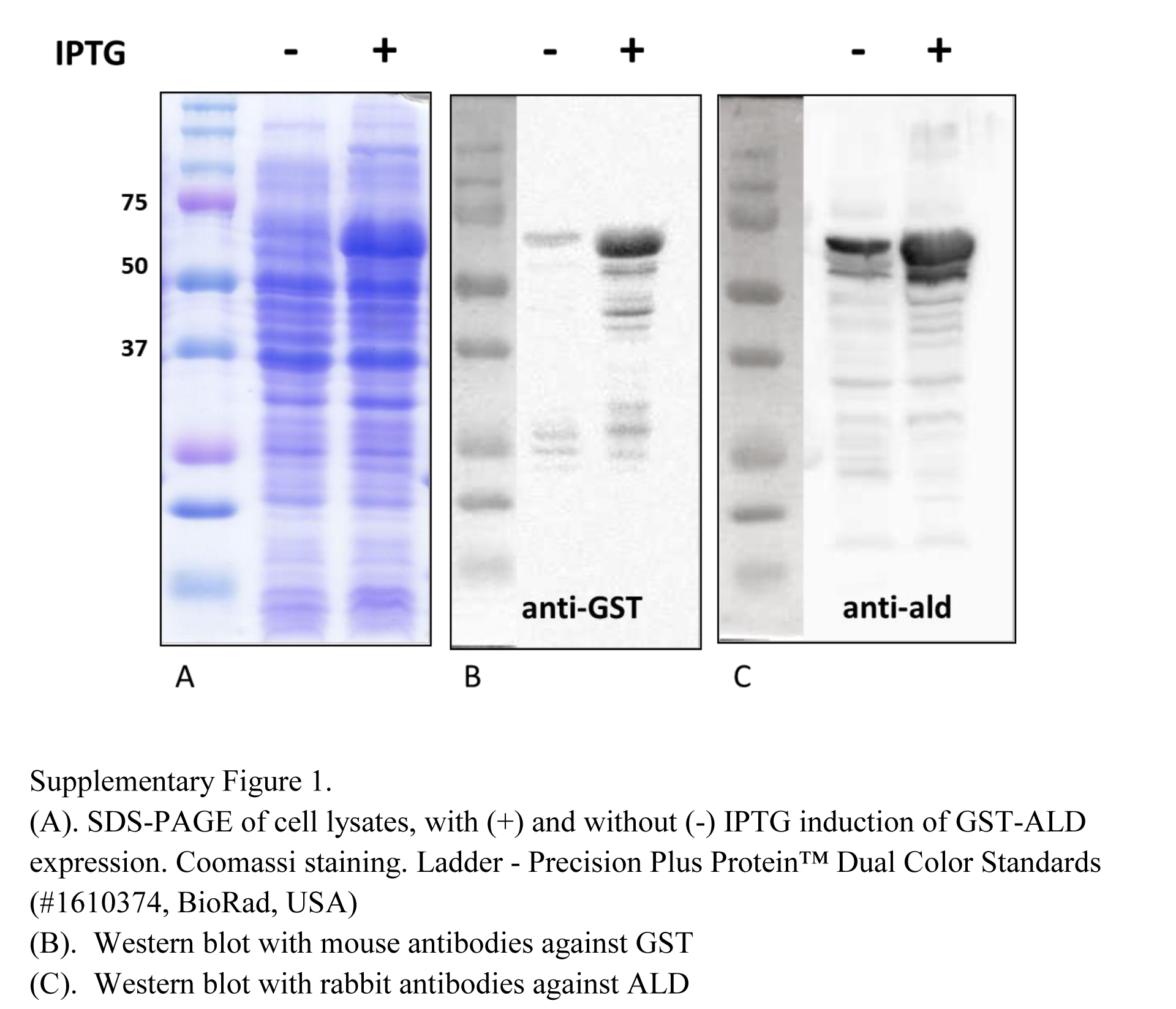

Supplement: Supplementary file 2 [file Image_1.tif]
