# Supplementary material for: A New Antisense Phosphoryl Guanidine Oligo-2′-O-Methylribonucleotide Penetrates Into Intracellular Mycobacteria and Suppresses Target Gene Expression
Source: Front Pharmacol. 2019 Sep 19;10:1049. doi: 10.3389/fphar.2019.01049 (PMC6778816; doi:10.3389/fphar.2019.01049)
Supplement: Supplementary file 3 [file Image_2.pdf]

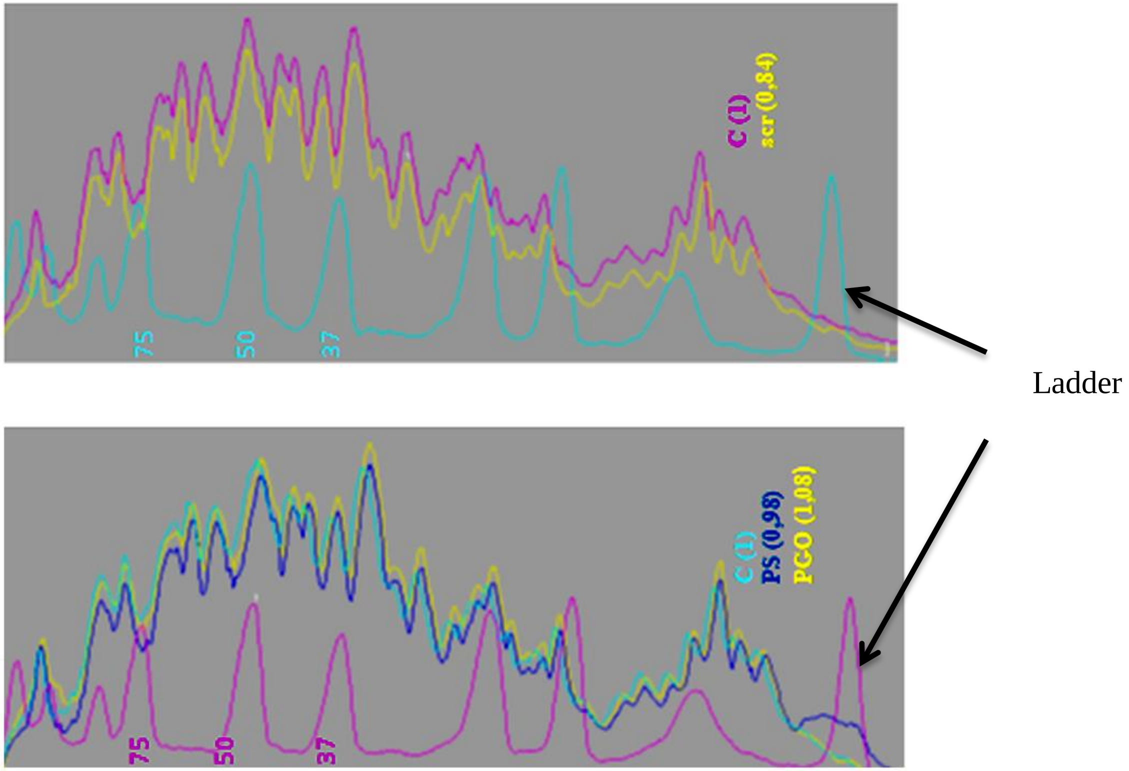

Supplementary Figure 2. Densitometry analysis of an SDS-PAGE gels.
The proteins were visualized by Coomassi staining, and analyzed by densitometry using Gel Pro Analysis software. Total protein amount in a lane is given as a relative density unit. The “Ladder” profile stands for Precision Plus Protein™ Dual Color Standards (#1610374, BioRad, USA).
