# Supplementary material for: A New Antisense Phosphoryl Guanidine Oligo-2′-O-Methylribonucleotide Penetrates Into Intracellular Mycobacteria and Suppresses Target Gene Expression
Source: Front Pharmacol. 2019 Sep 19;10:1049. doi: 10.3389/fphar.2019.01049 (PMC6778816; doi:10.3389/fphar.2019.01049)
Supplement: Supplementary file 4 [file Table_1.docx]

**Table 1. Oligonucleotides used in the study**

| **Name** | **Oligonucleotide sequence, 5’–3’***^a^* | | **Molecular mass** | |
| --- | --- | --- | --- | --- |
|  |  |  | **calcd** | **found** |
| ald-RNA | AGG AGC GAU CAU GCU CGU CGG AA | | – *^b^* | – *^b^* |
| ald | ttc cga cga gca tga tcg ctc ct | | – *^b^* | – *^b^* |
| ald-OMe | UUC CGA CGA GCA UGA UCG CUC CU | | – *^b^* | – *^b^* |
| ald-PS | t^S^*^c^*t^S^c^S^ c^S^g^S^a^S^ c^S^g^S^a^S^ g^S^c^S^a^S^ t^S^g^S^a^S^ t^S^c^S^g^S^ c^S^t^S^c^S^ c^S^t | | – *^b^* | – *^b^* |
| µ-ald | t^µ^*^d^*t^µ^c^µ^ c^µ^g^µ^a^µ^ c^µ^g^µ^a^µ^ g^µ^c^µ^a^µ^ t^µ^g^µ^a^µ^ t^µ^c^µ^g^µ^ c^µ^t^µ^c^µ^ c^µ^t | | 8974.2*^e^* | 8973.5*^e^* |
| ald-PGO | U**^f^*U*C*C*G*A*C*G*A*G*C*A*U*G*A*U*C*G*C*U*C*C*U | | 9691.3 | 9685.7 |
| scr-PGO | G*U*C*C*A*G*C*C*C*C*A*U*G*G*Aр | | 6563.3 | 6563.6 |
| FAM-PGO | U*U*C*C*G*A*C*G*A*G*C*A*U*G*A*U*C*G*C*U*C*C*U-FAM | | 10258.8 | 10255.6 |
|  | | | | |
| **Primers for qPCR** | | | | |
| Ald-smegm-for | | cctaccacctcatgcgcag | | |
| Ald-smegm-rev | | tgaccgcttcttcgagttcg | | |
| 16S-smegm-for | | atgtcggttcccttgtggc | | |
| 16S-smegm-rev | | caagggttgcgctcgttg | | |

*^a^* Ribonucleotides and 2’-O-methylribonucleotides are in uppercase, ribonucleotides are underlined; deoxynucleotides are in lowercase; *^b^* Obtained from a commercial supplier; *^c^* (^S^) – phosphorothioate linkage; *^d^* (^µ^) – mesyl phosphoramidate linkage; *^e^* Containing 5’-dimethoxytrityl (DMTr) group; *^f^* (*) – 1,3-dimethylimidazolidine-2-imino group; р – 6-aminohexyl phosphoryl guanidine group; FAM – 6-fluorescein carboxamide residue
